# Stem cells and beta cell replacement therapy: a prospective health technology assessment study

**DOI:** 10.1186/s12902-018-0233-7

**Published:** 2018-01-30

**Authors:** Klemens Wallner, Rene G. Pedroza, Isaac Awotwe, James M. Piret, Peter A. Senior, A. M. James Shapiro, Christopher McCabe

**Affiliations:** 1 Department of Emergency Medicine Research Group, Department of Emergency Medicine, University of Alberta, 8303 - 112 Street, Edmonton, AB T6G 2T4 Canada; 2 Michael Smith Laboratories and Department of Chemical & Biological Engineering, University of British Columbia, 2185 East Mall, Vancouver, BC V6T 1Z4 Canada; 3 Clinical Islet Transplant Program, Alberta Diabetes Institute, University of Alberta, 2000 College Plaza, 8215 - 112 Street, Edmonton, AB T6G 2C8 Canada; 4 Department of Medicine, University of Alberta, Edmonton, Canada; 5 Department of Surgery, University of Alberta, Edmonton, AB Canada

**Keywords:** Type 1 diabetes, Stem cells, Medical device, Transplantation, Disease simulation, Cost optimization, Cost modeling, Health technology assessment, Early technology assessment, Health economics

## Abstract

**Background:**

Although current beta cell replacement therapy is effective in stabilizing glycemic control in highly selected patients with refractory type 1 diabetes, many hurdles are inherent to this and other donor-based transplantation methods. One solution could be moving to stem cell-derived transplant tissue. This study investigates a novel stem cell-derived graft and implant technology and explores the circumstances of its cost-effectiveness compared to intensive insulin therapy.

**Methods:**

We used a manufacturing optimization model based on work by Simaria et al. to model cost of the stem cell-based transplant doses and integrated its results into a cost-effectiveness model of diabetes treatments. The disease model simulated marginal differences in clinical effects and costs between the new technology and our comparator intensive insulin therapy. The form of beta cell replacement therapy was as a series of retrievable subcutaneous implant devices which protect the enclosed pancreatic progenitors cells from the immune system. This approach was presumed to be as effective as state of the art islet transplantation, aside from immunosuppression drawbacks. We investigated two different cell culture methods and several production and delivery scenarios.

**Results:**

We found the likely range of treatment costs for this form of graft tissue for beta cell replacement therapy. Additionally our results show this technology could be cost-effective compared to intensive insulin therapy, at a willingness-to-pay threshold of $100,000 per quality-adjusted life year. However, results also indicate that mass production has by far the best chance of providing affordable graft tissue, while overall there seems to be considerable room for cost reductions.

**Conclusions:**

Such a technology can improve treatment access and quality of life for patients through increased graft supply and protection. Stem cell-based implants can be a feasible way of treating a wide range of patients with type 1 diabetes.

**Electronic supplementary material:**

The online version of this article (10.1186/s12902-018-0233-7) contains supplementary material, which is available to authorized users.

## Background

Although islet cell transplantation is effective for treating certain type 1 diabetes patients, some hurdles are inherent to this and other donor-based transplantation methods [1–4]. Two hurdles are the limited graft supply and graft rejection. One solution for islet cell transplantation could be to move from donor-harvested to stem cell-derived transplant tissue. That could involve production of pancreatic progenitor cells from human embryonic stem (hES) cells. Using stem cells in general may have some advantages compared to current islet cell transplantation. These advantages include the potential of producing stem cells in large quantities thereby eliminating the cell supply problem and possibly reducing the treatment cost per patient.

Research in that area of treatment has advanced from proof-of-principle studies in animals, to establishing controllable cell manufacturing processes, and the first clinical trials in humans [5–15]. As of 2017 clinical trials are ongoing in Canada and the United States that use a thin removable device which is implanted under the skin [7, 16]. This device has hES cell-derived pancreatic progenitor cells within a casing to shield the tissue from the immune system [15]. Those cells are expected to mature to functional endocrine cells which secrete insulin in a glucose-dependent manner [9, 14–16]. Further improvement in protection of transplant tissue could increase its viability and reduce graft rejection. The long term goal of research into beta cell replacement therapy is to reverse diabetes and completely avoid the need for immunosuppressive medication.

In 2011 Weir et al. mention, “due to the need for beta cell replacement therapy, much work has been done in the past decade to generate beta cells from a variety of cell sources” [13]. However, these efforts have had mixed success. A major barrier has been in the ability to direct cell lines to differentiate towards an endocrine lineage. That process was very inefficient and most cell lines could not be used. Further, technologies used in the preservation of graftable cells, for example through cooling to very low temperatures, have advanced considerably but are still difficult and costly [17, 18]. Use of simpler preservation technologies makes cell tissue more perishable but experiences in standard donor-derived transplantation may point towards greater affordability of such techniques. Still, those barriers add to existing complexities associated with supply logistics, regulatory frameworks and scaling out production to multiple cell manufacturing sites [19].

Given those developments and findings, stem cell-based beta cell replacement therapy is a case study for the necessity of prioritizing research resources when researching new healthcare technologies. In our study we aimed to explore the circumstances under which a stem cell-based graft tissue would be cost-effective, given its effectiveness is comparable to state of the art islet transplantation aside from immunosuppression drawbacks. Our core question is if and how such a new transplant option for beta cell replacement has a chance of being cost-effective.

## Methods

**Fig. 1:**
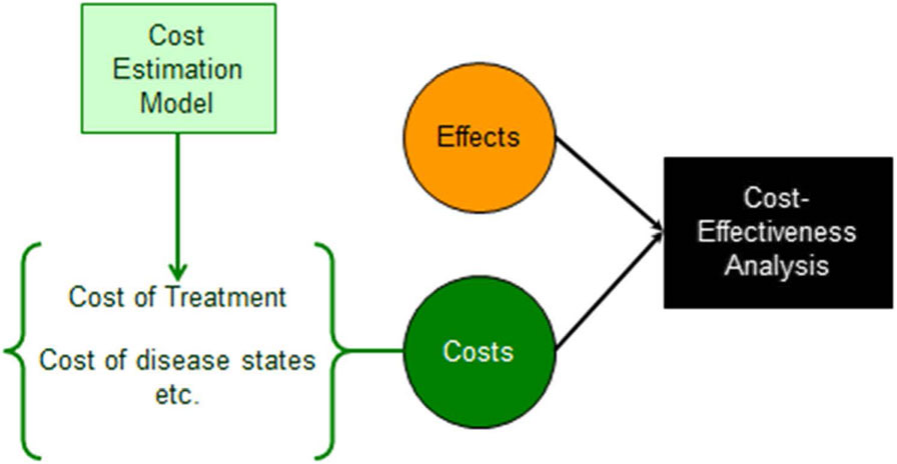
The role of our cost estimation model within the cost-effectiveness analysis. Note that we used the explicit cost optimization model for our stem cell-based treatment only

To model the cost of hES cell-derived transplant doses we used a two part cost-effectiveness and manufacturing model (Fig. 1). This stochastic model is based on a previous treatment model of type 1 diabetes [20] and the work by Simaria and colleagues [21]. Presuming equal effectiveness with the current technology islet transplantation, aside from the immunosuppression drawbacks, we then ran the model to simulate marginal differences in clinical effects and costs, between the new stem cell-based technology and our comparator intensive insulin therapy. We used the models outputs to estimate the cost-effectiveness of this trial-stage therapy.

### Compared treatments

The form of beta cell replacement therapy was modeled as a series of identical re-extractable subcutaneous implant devices (‘sheets’). Each of these devices contain hES-derived cells, specifically pancreatic progenitors which were modified to attain the function of beta cells. Those cells are enclosed within a casing which shields them from the immune system but allows the transport of nutrients to and hormones from the encapsulated cells [14, 15]. In our study we make the important assumption that this shielding effect completely removes the need for immunosuppressive medication. Patients in our study can get up to four transplantations [20]. The average proportion of patients with full graft function after each transplantation was assumed to be increasing from first to third and fourth transplantation, i.e. from 15% to 70% and then 85% for the third and fourth ones [20].

The comparator treatment is intensive insulin therapy involves frequent self-monitoring of blood glucose and multiple daily insulin injections, further details are described elsewhere [20].

### Cost-effectiveness analysis

We conducted a probabilistic and structural sensitivity analysis to investigate the cost-effectiveness of stem cell-based beta cell replacement therapy and to evaluate uncertainty around our results. Our simulation model was a discrete state-transition Markov model, which had a lifetime horizon. Its hypothetical cohort was composed of type 1 diabetes patients with hypoglycemia unawareness in the province of Alberta who fulfill the inclusion criteria to get an islet transplant. The model took the perspective of the provincial healthcare provider and its inputs were the same as in the pre-existing cost-effectiveness model [20], except for the described variations. Effectiveness was expressed in quality-adjusted life-years (QALYs) to measure the impact of therapy on both quality of life and life expectancy. All monetary estimates are expressed in 2016 Canadian dollars, with necessary adjustments made using the Canadian consumer price index for health and personal care [22, 23].

We updated model parameters from our pre-existing model of unstable type 1 diabetes [20] as described below. We model a future technology functioning without the need of immunosuppression. Therefore we had to change the parameters that – even partially - had to do with this medication. For that we removed all disutilities, costs and probabilities that had only to do with immunosuppression. The parameters for rate, costs and disutility of initial complications were adjusted by lowering each by 40% because this portion was attributed solely to immunosuppression. Specifically the rate changed from 0.65 to 0.39, the cost from $600 to $360, and the disutility from 0.05 to 0.03. Further, a study of the impact of type 1 diabetes complications (*N* = 2341) served us to update our utility i.e. quality of life estimates [24]. Patients in that study were more comparable to our hypothetical cohort than the ones in our original data sources. Yet they were still younger (39.3 vs. 47.0 years old) and with shorter diabetes duration (16.3 vs. 29.4 years) [20, 24]. Given that new evidence, we included neuropathy in the diabetes-related complications and adjusted our overall estimate for the complications state not only for multiple complications, but also to fit the actual age and duration of diabetes in our cohort [2, 24–29]. The utility parameter in the complications state was therefore adjusted from 0.57 to 0.47.

The cost-effectiveness model was constructed and run with the software TreeAge Pro 2016 (Williamstown, MA, USA). The cost of goods modeling was constructed and run using Microsoft Excel. Costs and benefits were discounted at 3%, and sensitivity analysis were performed at 0% and 5%. These were the rates that had been recommended by the Canadian Agency for Drugs and Technologies in Health [30]. Half-cycle correction was applied. Our probabilistic analyses used 64,000 iterations for each scenario. We estimated the value of further research reducing the decision uncertainty by way of value-of-information analysis [31, 32]. We calculated the expected value of perfect information (EVPI) and the expected value of partial perfect information (EVPPI) for the cost of goods group of parameters. For that EVPPI calculation we used nested Monte Carlo simulations with 600 ‘outer’ and 600 ‘inner’ loops. Additional information on our value of information approach, including choice of WTP thresholds, can be found elsewhere [20].

### Integration of cost of goods results

To integrate the results of the cost of goods modeling into our cost-effectiveness model, we took the parameter representing the cost per transplantation within the transplant state in the original model, and split it up into non-dose costs and dose costs. Based on literature we assumed the non-dose costs, including the transplant procedure, to be about 38% of the costs per transplantation [33]. For that parameter of our model we used a Gamma distribution with a relative standard deviation of 10% (i.e. standard deviation as percentage of the mean). For the dose costs, based on our cost of goods (COG) model we used the following equation:

1 $$ Dose\kern0.17em costs={\mathrm{COG}}_{\mathrm{upstream}}\times {\mathrm{factor}}_{\mathrm{COG}\;\mathrm{downstream}}\times {\mathrm{factor}}_{\mathrm{additional}\kern0.17em \mathrm{regulation}} $$

**Fig. 2:**
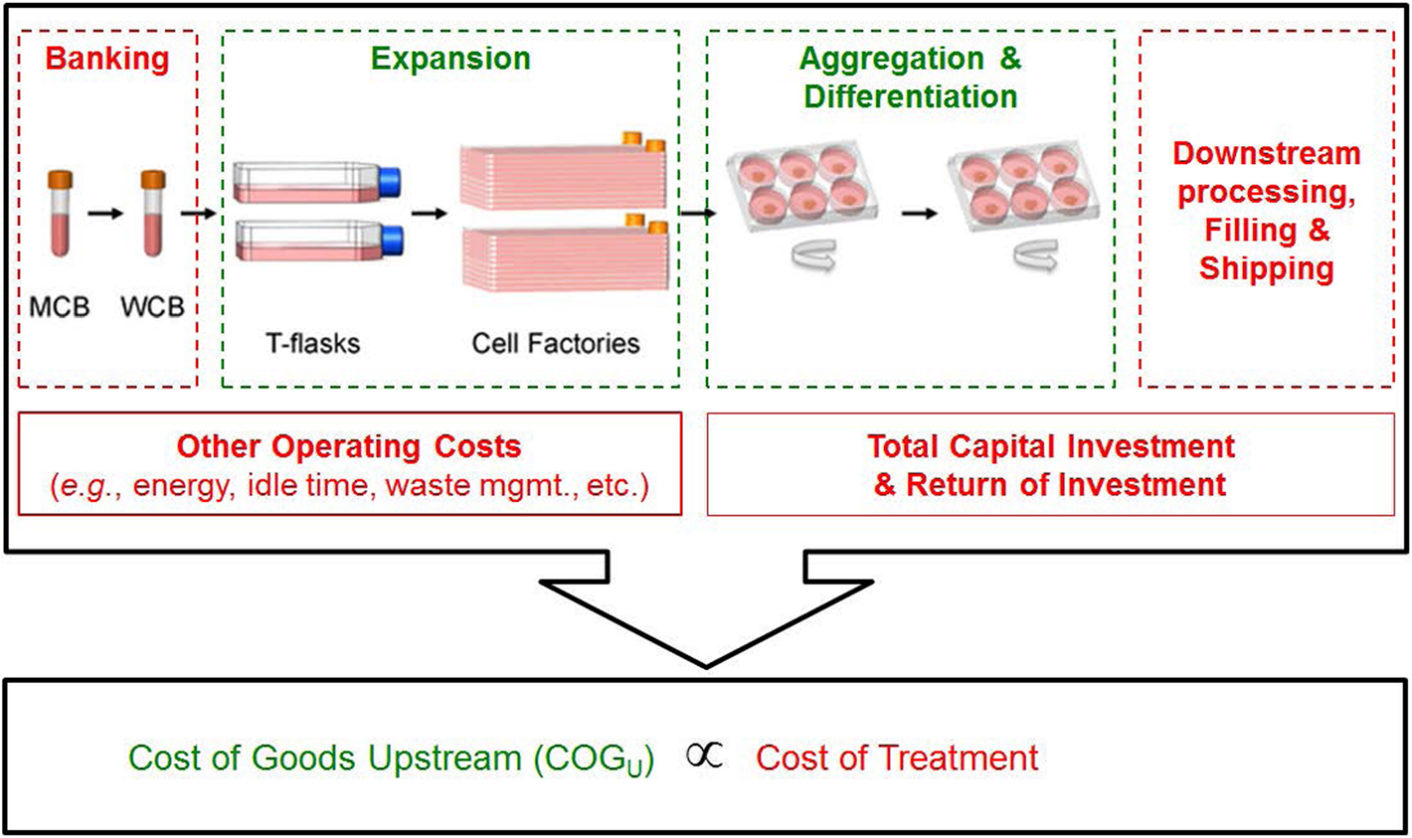
Illustration of cost of goods modeling in a biotechnology application. On the top one can see the different parts that compose the cost of goods for manufactured cell products. The bottom part portraits the upstream cost of goods, highlighted in green, as proportional to the overall cost of the treatment. That is a simplification compared to our analysis, which treats costs after cell product arrival at the hospital as independent from the cost of upstream cell processing

Here dose costs are calculated multiplying the cost of goods upstream by a cost of goods downstream factor and a factor that we called “regulatory burden factor”. The cost of goods upstream came from the above described fitted distributions estimating the “pure” production cost of the cells. The downstream factor accounted for the so-called downstream processing, which is necessary after the cells are produced, e.g. cell harvesting, volume reduction, washing, formulation for storage or delivery (see Fig. 2). We used the regulatory burden factor to account for the possibility of additional regulatory burden due to stricter regulatory requirements for a new cell production process whose product is designed to enter regular healthcare practice.

The mean of the cost of goods downstream factor (multiplier) was assumed to be three, four and eight, depending on scenario, based on expert opinion and published literature [34]. The mean of the regulatory burden factor was assumed to be 1.2 and 1.8 depending on scenario (i.e. 20% and 80% additional costs respectively due to regulation). Both multipliers were made probabilistic using a Log-normal distribution and expert opinion on variation estimates.

### Cost of goods modeling

We modeled the cost of goods in Microsoft Excel based on a report describing manufacture of pancreatic progenitors from single cell cultures of hES cells [6]. Briefly, the modeled process consisted of thawing one or more frozen hES cells vials and expanding them in adherent culture for about 14 days, passaging four times. Then, hES cells were cultured in suspension forming cell clusters, while a cocktail of molecular signals was added to the media to promote stepwise differentiation of hES cells into pancreatic progenitors.

The cost of goods was estimated by adapting a cost minimization decisional tool for this manufacturing process [21]. The tool selected the optimal set of disposable culture vessels for a user-specified annual demand, lot size, cell dose and user-specified manufacturing constraints, i.e. maximum allowed number of culture vessels per lot, which was set to 100. In its estimation process, the tool calculated the material (i.e. media, disposable culture vessels), labor, quality control and equipment costs involved in the expansion and differentiation stages of the process for a battery of sequential culture vessel combinations (see Fig. 2). Additional parameters utilized during the cost calculations were the overall yield of the manufacturing process and the expansion fold of the hES cells. In that way the upstream cost of goods were estimated.

The cost minimization decisional tool did not include the downstream component of the manufacturing process (e.g., finishing, packaging, shipping), therefore 95% credible ranges were derived for cost estimates in four different settings: two cell culture methods (adherent and suspension), and each with two supply levels (50 and 500 doses per year) (also see Table 1). The credible ranges were used to fit Gamma distributions, i.e. the lower and upper bounds of the credible ranges for every cost estimate were equated to the values of cumulative density functions (CDF) at values of CDF = 0.025 and CDF = 0.975. The distributions were then used directly in the health economic modeling software.

**Table 1:** Credible ranges and fitted distributions for cost of goods upstream

Production setting		Range		Gamma distribution			
Cell culture	Supply	Lower bound	Upper bound	Mean	RSD	Shape	Rate
Adherent	50	$21,300	$83,900	$47,443	34.00%	8.6505	0.0001823
Adherent	500	$14,700	$73,800	$38,585	39.60%	6.3769	0.0001653
Suspension	50	$16,900	$54,900	$33,193	29.42%	11.5535	0.0003481
Suspension	500	$10,300	$53,100	$27,535	40.20%	6.1880	0.0002247

### Scale of manufacturing

We simulated four manufacturing modes: local production (e.g. at one University only), large scale production (one central lab produces all the doses and then ships them to the hospitals), and two scale-out production modes (local and large scale). The scale-out scenarios involved a network of several labs producing their own doses at their respective location but collaborating with each other through sharing expertise and research resources. We simulated one scale-out scenario for local productions and one for large scale productions. The local and large scale production scenarios assume a demand of 50 and 500 doses per year respectively. In general, the scale out approach may engage the capabilities of multiple local institutions and companies. It could, however, also contribute to unequal product quality and an increased overhead costs.

We estimated the long-term capacity to perform device implants in Canada to be 10 clinical centers. That estimate was derived by counting the hospitals on the list of transplant centers by the Canadian Organ Replacement Register in which clinicians performed islet cell transplants or other transplants of at least three different kinds of organs [35, 36]. We took this as clinical capacity to carry out transplantations of beta cell replacement devices that *do not* require immunosuppression. In the short term there could be two centers, one for Western Canada and one Eastern Canada.

We describe the demand for and composition of the doses of beta cell replacement tissue as follows. The annual demand of beta cell replacement doses was based on the current number of islet cell transplants in Canada and assumed to be 50 per transplant center, which was derived as linear extrapolation of transplant numbers in at the University of Alberta Hospital. Further we presumed the number of lots produced per year is 10, i.e. about one per month, and a minimum of 500 million cells are required per dose. Those numbers were derived from considerations of cell quality loss over time and the production figures above. Based on experience in the biotechnology sector the production assumed one of two production technologies, adherent or suspension cell culture approach, each with optimized production set ups for the two demand options (50 or 500 doses per year).

As a substantial simplification due to the novelty of the membrane technology, we presumed the cost of the device casing without the cells is off-set by reductions in costs through increased ability to plan transplantation times and processes.

## Results

Our analysis shows that the use of stem cells for beta cell replacement therapy can be an effective use of health budget funds. However, there is substantial uncertainty around the costs of this technology. We calculated the expected range of treatment costs for hES cell-based beta cell tissue. Our probabilistic results indicate that currently this technology could be cost-effective at a WTP threshold of $100,000 per QALY because three scenarios have ICERs substantially below that threshold (Tables 2 and 3). Specifically the ICERs of scenarios Adh20, Sus19 and Sus20 are $79,230, $89,173 and $60,111 per QALY respectivly. For the 95% Confidence interval values around our results please see in Additional file 1.

**Table 2:** Results for different scenarios using adherent cell culture (means per patient)

Scenario						Cost		Benefit		ICER	EVPI		Maximum Partial EVPI Dose Costs
Index	Production mode	Supply per facility	COGd factor	Regulatory factor	Variation (RSD^a^)	Strategy	Difference	Strategy	Difference		WTP per QALY		
											$50,000	$100,000	
Scenarios with 3% discount rate													
Comp1	(Comparator 3%)					74,230		11.12					
Adh1	Local	50	4	1.2	22.5%	629,181	554,951	13.85	2.73	203,203	18	4220	90,957
Adh2	Local	50	4	1.2	50.0%	628,936	554,707	13.85	2.73	203,114	677	19,749	135,128
Adh3	Local	50	4	1.8	22.5%	876,810	802,580	13.85	2.73	293,877	2	721	143,704
Adh4	Local	50	4	1.8	50.0%	873,510	799,281	13.85	2.73	292,669	169	8061	214,930
Adh5	Scale out local	50	3	1.2	22.5%	504,903	430,673	13.85	2.73	157,697	87	11,725	69,691
Adh6	Scale out local	50	3	1.2	50.0%	504,835	430,606	13.85	2.73	157,673	1493	32,911	106,144
Adh7	Scale out local	50	3	1.8	22.5%	690,050	615,819	13.85	2.73	225,492	11	2623	102,737
Adh8	Scale out local	50	3	1.8	50.0%	688,524	614,294	13.85	2.73	224,933	432	15,297	167,801
Adh9	Scale out local	50	8	1.8	22.5%	1,616,386	1,542,156	13.85	2.73	564,685	0	19	273,576
Adh10	Scale out local	50	8	1.8	50.0%	1,606,953	1,532,722	13.85	2.73	561,231	9	1052	443,892
Adh11	Large scale	500	4	1.2	22.5%	536,915	462,685	13.85	2.73	169,420	127	11,621	78,153
Adh12	Large scale	500	4	1.2	50.0%	536,730	462,501	13.85	2.73	169,351	1501	31,043	124,247
Adh13	Large scale	500	4	1.8	22.5%	738,478	664,248	13.85	2.73	243,225	24	3085	117,352
Adh14	Large scale	500	4	1.8	50.0%	736,541	662,311	13.85	2.73	242,516	499	14,700	192,416
Adh15	Scale out large	500	3	1.2	22.5%	435,777	361,548	13.85	2.73	132,386	453	24,792	63,732
Adh16	Scale out large	500	3	1.2	50.0%	435,661	361,432	13.85	2.73	132,344	3005	47,591	96,481
Adh17	Scale out large	500	3	1.8	22.5%	586,704	512,474	13.85	2.73	187,650	82	8143	93,084
Adh18	Scale out large	500	3	1.8	50.0%	585,166	510,936	13.85	2.73	187,088	1118	25,291	148,572
Scenarios with 0% discount rate													
Comp2	(Comparator 0%)					113,175		16.09					
Adh19	Local	50	4	1.2	22.5%	663,514	550,339	20.60	4.51	122,159	1395	52,620	90,906
Adh20	Scale out large	500	3	1.2	22.5%	470,111	356,936	20.60	4.51	79,230	11,315	30,540	63,752
Scenarios with 5% discount rate													
Comp3	(Comparator 5%)					58,559		9.09					
Adh21	Local	50	4	1.2	22.5%	616,693	558,134	11.18	2.09	267,339	0	614	90,973
Adh22	Scale out large	500	3	1.2	22.5%	423,290	364,731	11.18	2.09	174,701	32	6396	63,730

All scenarios used the base case assumptions with the described structural deviations. Cost measure is Canadian dollar (2016). Benefit measure is QALY. All result numbers are rounded and including sampling variation

^a^Relative standard deviation (RSD; i.e. SD as percentage of the mean) that was assumed for the two factors

**Table 3:** Results for different scenarios using suspension cell culture (means per patient)

Scenario						Cost		Benefit		ICER	EVPI		Maximum Partial EVPI Dose Costs
Index	Production mode	Supply per facility	COGd factor	Regulatory factor	Variation (RSD^a^)	Strategy	Difference	Strategy	Difference		WTP per QALY		
											$50,000	$100,000	
Scenarios with 3% discount rate													
Comp1	(Comparator 3%)					74,230		11.12					
Sus1	Local	50	4	1.2	22.5%	480,575	406,346	13.85	2.73	148,790	56	12,126	59,158
Sus2	Local	50	4	1.2	50.0%	479,911	405,680	13.85	2.73	148,546	1541	35,232	92,836
Sus3	Local	50	4	1.8	22.5%	654,137	579,906	13.85	2.73	212,342	4	2464	88,524
Sus4	Local	50	4	1.8	50.0%	651,401	577,171	13.85	2.73	211,340	450	16,335	141,768
Sus5	Scale out local	50	3	1.2	22.5%	393,796	319,566	13.85	2.73	117,014	305	28,627	47,474
Sus6	Scale out local	50	3	1.2	50.0%	393,094	318,864	13.85	2.73	116,757	3215	53,937	76,931
Sus7	Scale out local	50	3	1.8	22.5%	523,705	449,475	13.85	2.73	164,582	33	8084	66,874
Sus8	Scale out local	50	3	1.8	50.0%	521,437	447,207	13.85	2.73	163,752	1084	28,588	113,389
Sus9	Scale out local	50	8	1.8	22.5%	1,172,878	1,098,648	13.85	2.73	402,287	0	50	169,848
Sus10	Scale out local	50	8	1.8	50.0%	1,163,974	1,089,744	13.85	2.73	399,026	35	2719	295,702
Sus11	Large scale	500	4	1.2	22.5%	421,724	347,494	13.85	2.73	127,240	590	28,370	59,316
Sus12	Large scale	500	4	1.2	50.0%	420,338	346,108	13.85	2.73	126,733	3399	51,260	83,398
Sus13	Large scale	500	4	1.8	22.5%	565,342	491,112	13.85	2.73	179,828	116	9785	86,421
Sus14	Large scale	500	4	1.8	50.0%	562,360	488,130	13.85	2.73	178,736	1294	27,942	124,381
Sus15	Scale out large	500	3	1.2	22.5%	349,649	275,419	13.85	2.73	100,848	1666	43,136	47,205
Sus16	Scale out large	500	3	1.2	50.0%	349,048	274,819	13.85	2.73	100,629	6192	64,312	64,826
Sus17	Scale out large	500	3	1.8	22.5%	457,207	382,977	13.85	2.73	140,232	384	21,505	67,653
Sus18	Scale out large	500	3	1.8	50.0%	455,948	381,718	13.85	2.73	139,772	2669	43,714	101,004
Scenarios with 0% discount rate													
Comp2	(Comparator 0%)					113,175		16.09					
Sus19	Local	50	4	1.2	22.5%	514,909	401,734	20.60	4.51	89,173	4389	40,830	59,131
Sus20	Scale out large	500	3	1.2	22.5%	383,981	270,808	20.60	4.51	60,111	26,451	7684	47,205
Scenarios with 5% discount rate													
Comp3	(Comparator 5%)					56,558		9.09					
Sus21	Local	50	4	1.2	22.5%	468,087	409,529	11.18	2.09	196,160	1	2042	59,162
Sus22	Scale out large	500	3	1.2	22.5%	337,161	278,602	11.18	2.09	133,447	172	16,410	47,207

All scenarios used the base case assumptions with the described structural deviations. Cost measure is Canadian dollar (2016). Benefit measure is QALY. All result numbers are rounded and including sampling variation

^a^Relative standard deviation (RSD; i.e. SD as percentage of the mean) that was assumed for the two factors

However, the results also indicate that large-scale production has the best chance of providing affordable graft tissue, as can be seen in scenarios ADh15, Adh16 and Adh20 in Table 2. These scenarios have the highest value for money for this method of cell culture. That means that for a given patient benefit the costs are minimized. For the suspension cell cultures the same scenarios also had the lowest ICERs (see scenarios Sus15, Sus16 and Sus20 in Table 3).

With adherent cell culture all scenarios have ICERs higher than $100,000 except scenario ‘Adh20’, which has a 0% discount rate and a supply of 500 doses per year. On the other side all suspension cell culture scenarios also have ICERs higher than $100,000 except for the scenarios ‘Sus19’ and ‘Sus20’, both use a 0% discount rate. Such a low discount rate does value small benefits with a long duration more favorable than a higher discount rate would.

**Fig. 3:**
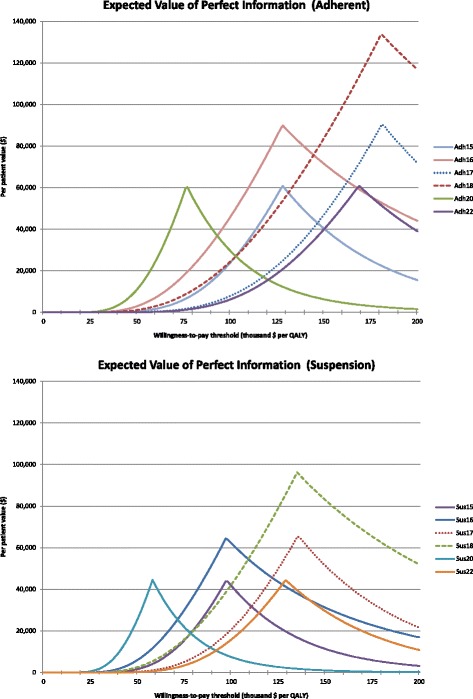
Expected value of perfect information results. Displayed are results of the value of further research for the scenarios using adherent (top) and suspension (bottom) cell culture techniques. All values are per patient calculations for willingness-to-pay thresholds of up to $200,000 per additional QALY. One can see the different values between cell culture techniques and between production scenarios within each technique. The dotted lines represent scenarios presuming an 80% increase of costs due to additional regulatory requirements compared to regulations currently in place. Scenarios Adh 20 and Sus 20 use a 0% discount rate

Our finding that use of stem cells for beta cell replacement therapy can be an effective use of health budget funds can be confirmed by the value of information results. The value of information can be seen as both a measure of decision uncertainty as well as an indicator of research investment value [31, 32]. In Fig. 3 we show the expected value of research into the cost-effectiveness of the technologies under consideration. One can see all per-patient EVPI values do peak at high cost-effectiveness thresholds but there also is considerable value when using for instance a $50,000 threshold. That means that further research into the cost-effectiveness of this treatment can be worthwhile for Alberta up to these upper limits per patient, even if one uses a strict cost-effectiveness threshold of $50,000.

We found uncertainty around the mean outcomes and therefore the need to conduct further research in this kind of disease treatment. This becomes clear when we consider the results in Fig. 3 and the number of patients that could benefit. In Alberta alone there are more than 4000 patients with unstable type 1 diabetes [37–42]. Extrapolating this estimate, one can expect to have about 500,000 patients in North America [37–43]. When comparing those figures with the per patient values in Fig. 3, one can argue that further research in this area of technology can be a sound investment of health budget funds.

We report the treatment dose costs with the production settings we used for a set of example regulatory and cost of goods downstream factors (Table 4). In this comparison one can see the adherent cell culture with 50 dose per year setting has on average no chance of being cost effective because its mean is much higher than any of the maximum costs. The ‘adherent 500’ setting can only be cost effective with a 1% (or lower) discount rate, without immunosuppression and only at a less strict threshold of $100,000. At that threshold and discount rate both the suspension cell culture settings can be cost effective without immunosuppression.

**Table 4:** Full dose costs using example cost of goods downstream and regulator factors

Production setting		Factors		Full dose costs^a^		
Cell culture	Supply	Cost of goods downstream	Regulatory	Mean	Lower range	Upper range
Adherent	50	3	1.2	$170,795	$76,680	$302,040
Adherent	500	3	1.2	$138,906	$52,920	$265,680
Suspension	50	3	1.2	$119,495	$60,840	$197,640
Suspension	500	3	1.2	$99,126	$37,080	$191,160

^a^Means and values at the lower and upper 95% credible range

We report the full dose costs with the production settings we used for a set of example regulatory and cost of goods downstream factors (Table 4). In this comparison one can see that our results point towards an increased efficiency through a) high volume production, and b) use of adherent cell culture technique.

## Discussion

Our results show that the use of stem cells for beta cell replacement therapy can be an effective use of health budget funds. Still, there is substantial uncertainty around the costs of this technology. Both of those findings confirm that methods of cost modeling combined with value-of-information analysis can be useful tools for aiding the prioritization. This especially applies to new healthcare technologies. Estimating the cost of transplant tissue we found it possible for the treatment to be cost-effective at commonly used cost-effectiveness thresholds if it greatly reduces the need for immunosuppression. The value of information as well as other results depend very much on the assumptions in the respective scenarios. Those assumptions include transplantations costs, especially transplant tissue cost of goods and immunosuppression, as well as discount rates.

In near future stem cell therapy could be expanded to a much broader population of type 1 diabetes patients. Currently the expansion of beta cell replacement therapy in general is limited by organ supply and risks of immunosuppression. If outcomes were better than for islet transplantation, i.e. long term euglycemia and insulin independence, the lifetime costs of conventional therapy due to management of diabetes complications would be avoided. That would include costs not considered in this analysis which would fall outside of the budget of the provincial health care service, e.g. costs covered by the federal health budget or costs for the patient’s family or private insurance.

### Challenges of donor-harvested transplants in Canada

Additional challenges of the donor-harvested approach in Canada could lead there to more readiness to adopt a stem cell based therapy approach even with initially higher costs. Among those challenges one needs to consider the relative shortage of organ donations, combined with great geographic distances between donors and the islet processing and transplantation site [44]. These factors can lead to two kinds of costs of timely organ transport. The monetary costs are sometimes covered by different regional health care services or air lines. Air transport companies are known to occasionally ship donor organs free of charge. Nevertheless non-monetary costs are unavoidable. An example is the cost of organ deterioration from with progressive cold ischemia can mar graft yield later on.

All those costs tend to be less for more densely populated countries, or even regions with different organ donation legislation, which can make a considerable difference in donor availability [45]. International coordination of donor organ availability could also further increase the efficient use available clinical resources. However, such coordination tends to come with substantial political and practical complexities, which require further research but are beyond the topic of this study.

### On efficient treatment delivery

While high volume implant production can theoretically be cheaper one needs to weigh that with several considerations regarding demand, clinical capacity and other practical limitations. One of those considerations is that stem cell-derived doses are currently as perishable as the donor derived cells. This means they have to be used within about 12–36 h of completion of the production process.

The difference between stem cell derived tissue and harvested cells is here that one can determine the time when the tissue is ready. Instead of the cell dose coming into the hospital more or less randomly at any time of the day or night, one can time the production process so that the dose or doses arrive at the hospital at a predefined day and time of the day.

In that way one can avoid the additional costs involved in nightly or short notice transplantations. But graft doses still have to be transplanted as quickly as possible. If several doses arrive at the same time it is also the case that all need to be transplanted within a short period of time. That could be accomplished if for example every week or every month 10 doses arrive at a hospital and are then all transplanted into 10 patients within the same day.

For that reason the number of lots produced per year is important. Every time a lot is produced all the doses of the lot have to be used within about one day or else go waste. That is because currently it is not possible to preserve beta cell progenitors over long periods, e.g. via cryo-preservation. In this context, it is advantageous from an economic perspective to produce several lots per year with smaller lot sizes, since it is impossible to transplant for example 500 doses in one day – even if spread over 10 transplantation centers.

Given the nature of the cells, transporting the patients to a central location – as is done currently - might be a better idea than transporting the cells to multiple patient hospitals across Canada. That is because transport of patients may actually be more affordable than the sum of: a) the health lost through the certain quality loss in the highly perishable cells through transport duration, b) the monetary costs from transporting the cells on a punctual just-in-time basis, c) the costs of duplication of in-hospital infrastructure and staff training.

### Limitations

Ongoing breakthroughs for example in current good manufacturing practice (cGMP) and mass cell expansion and limit the longevity of our estimates and modeling efforts. Breakthroughs include the genetic engineering technique CRISPR (Clustered Regularly Interspaced Short Palindromic Repeat) and the use of modern bioreactors which aid various kinds of bioprocessing [46, 47]. All those technologies have great influence on the capacities of researchers to generate new or more affordable ways of producing transplantable tissue.

We acknowledge that intensive insulin therapy as only comparator strategy to stem cell-based beta cell replacement therapy did limit the scope of our results. However, we consider the comparator and hypothetical patient cohort in our model to be appropriate because of the patient population under consideration. We explicitly limit our study population to patients who: 1) do not have the degree of major comorbidities which would justify risks of whole organ transplantation, 2) are on intensive insulin treatment and 3) are candidates for islet transplantation. Still, we think further studies in a North American context do need to include donor-base islet transplantation as one of the comparators. Other donor-based approaches to address type 1 diabetes could also be integrated.

The cost of the semi-permeable membrane in which the cells are enclosed had to be estimated doe to lack of data. This and the fact that we did not change the follow up costs and frequency compared to the study on islet transplantation are clear limitations of this study. However, in light of the technology under consideration being new and containing living cell tissue, the differences between the actual and our estimated follow-up costs are likely smaller than for a less complex implant device.

We expect that implantation would likely be an outpatient procedure with much more limited risks compared to islet transplantation, or even whole organ transplantation. In this study we presumed the new treatment technology would still be require an in-patient procedure including four days of hospital stay. Compared to that estimate, an out-patient procedure would further reduce the costs of stem cell-based beta cell replacement therapy while increasing patient quality of life.

One of the main goals of using re-extractable sheets for transplantation, instead of the standard cell injection into the liver, is to shield the cells from being attacked by the immune system. Since this is an early health technology assessment of a very new technology we made the assumption that this goal can be achieved without the use of imunosuppressive drugs. If future developments show that this is not the case then immunosuppression would be necessary and with it would come the usual costs and side effects as mentioned elsewhere [20].

## Conclusions

Using new grafts substantially increased the value of research into beta cell replacement therapy, especially when also addressing the need for immunosuppression. Such a technology can improve treatment access and quality of life for patients through increased graft supply and protection. Stem cell-based implants can be a feasible way of treating a wide range of patients with type 1 diabetes.

## Additional file

Additional file 1: 95% Confidence Interval of Results. We report the 95% confidence interval for the costs and benefits of all our scenarios and the ICERs that were calculated from those values. (PDF 60 kb)

## Abbreviations

cGMP: Current good manufacturing practice
COG: Cost of goods
CRISPR: Clustered Regularly Interspaced Short Palindromic Repeat technology, a genome editing tool
EVPI: Expected value of perfect information
EVPPI: Expected value of partial perfect information
hES cells: Human embryonic stem cells
ICER: Incremental cost-effectiveness ratio
QALY: Quality-adjusted life-year
RSD: Relative standard deviation
SD: Standard deviation
Wtp: Willingness to pay per additional QALY
