# Supplementary material for: Stem cells and beta cell replacement therapy: a prospective health technology assessment study
Source: BMC Endocr Disord. 2018 Jan 30;18:6. doi: 10.1186/s12902-018-0233-7 (PMC5791348; doi:10.1186/s12902-018-0233-7)
Supplement: Additional file 1: — 95% Confidence Interval of Results. We report the 95% confidence interval for the costs and benefits of all our scenarios and the ICERs that were calculated from those values. (PDF 60 kb) [file 12902_2018_233_MOESM1_ESM.pdf]

$$\textit{Dose costs} = \mathrm{COG_{upstream}} \times \mathrm{factor_{COG\ downstream}} \times \mathrm{factor_{additional\ regulation}} \qquad (1)$$
